# Surging trends of infertility and its behavioural determinants in India

**DOI:** 10.1371/journal.pone.0289096

**Published:** 2023-07-25

**Authors:** Sampurna Kundu, Balhasan Ali, Preeti Dhillon

**Affiliations:** 1 Centre of Social Medicine and Community Health, Jawaharlal Nehru University, New Delhi, India; 2 Department of Survey Research & Data Analytics, International Institute for Population Sciences, Mumbai, Maharashtra, India; Istanbul University-Cerrahpasa Faculty of Health Sciences, TURKEY

## Abstract

The World Health Organisation (WHO) has recognised infertility as a public health issue. Although biological factors are considered to be the primary cause, factors like social, health, and lifestyle factors can all have an adverse effect on a couple’s ability to reproduce. The study aimed to comprehend the infertility scenario in India and explore some of the potential causes. The study used standard demographic definitions and four rounds of the National Family Health Survey (NFHS) from 1992–1993 to 2015–16 to estimate the levels of primary and secondary infertility in India. Bivariate analysis, the t-test, and the Chi-square test were applied to capture significant changes in infertility over time. The multivariate logistic regression model was used to understand the extent of infertility among Indian couples from various socioeconomic groups, lifestyle levels, and reproductive behaviour in 2015–16. Primary infertility declined steadily from 1992 to 2015, whereas secondary infertility increased from 19.5% in 1992–93 to 28.6% in 2015–16. This trend is related to declining fertility rates, particularly in India’s southern states. Age at marriage, biological factors, and lifestyle factors were all strongly linked to infertility. People with higher education levels and late marriages were more likely to experience primary infertility. Alcohol consumption, smoking, obesity, and noncommunicable disease are all strongly linked to secondary infertility. Our study has policy implications, and we draw attention to alarming infertility in India, which has gone unnoticed due to large population. We suggests enhancing the current health and reproductive programmes, educating people about improving their lifestyle choices and sexual behaviour, and calling attention to a significant shift in fertility dynamics.

## Introduction

Infertility is a global reproductive issue that affects both males and females, but it is rarely discussed in public and is often overlooked. It has been neglected in the context of a health issue, and it is also a topic for social science research in South Asia, or more broadly in the developing world (1). In 1994, the Program of Action of the International Conference on Population and Development (ICPD) in Cairo, provided more explicit recognition of infertility as a health priority. The reproductive and child health programmes, however, do not adequately address the infertility issues in India. Infertility is a serious and alarming health issue with ramifications for both social and physical well-being. Parenthood is a highly emphasised and nearly vital event for couples in every society. Literature suggested that the consequences of infertility will be larger in those countries where pro-natal culture exists [1]. In these countries, motherhood is considered as a vital event and one of the most desirable goals for couples, especially women. It is seen as natural and related to feminine identity. In every society, children are traditionally expected to care for and support the elderly. Further, a few studies found that children and family care are necessary even in those societies which has a good social support system and are expected to provide care, mental, economic and environmental support to the elderly [2]. Childlessness has its own social, psychological, cultural, religious, and economic consequences and couples themselves consider childlessness as a lifetime tragedy [3]. However, women suffer to a great extent from childlessness as compared with men, and they always bear the burden of psychological, family and community pressure [3–5]. For many couples, childlessness is a tragedy because all couples who desire a child, do not experience parenthood. Infertile couples experience a sense of failure and exclusion due to expectations of society, family, faith, and culture [5].

The world health organisation (WHO) has recognised infertility as a public health issue [6]. Infertility is considered a personal problem of any couple, but it has a greater impact on women’s life. Evidence revealed that separation and divorce rate are much higher among infertile couples in African countries [7]. A few studies also documented domestic violence physical and psychological harassment by family members [8]. Studies from India suggested that Infertile couples are mostly stigmatised in society, leading to a sense of isolation and exclusion [3,5].

WHO suggests that worldwide about 8–12 per cent of couple suffer from infertility, and its incidence rate varies around the world [9]. The majority of couples who suffer infertility issues come from developing countries, and the highest infertility rates are reported from South and Central Africa [6,10,11]. Estimates of Census of India, 1981 portray that about 4–6 per cent of the couple were suffering from infertility [12]. The estimates of the Census of India (1981, 1991, 2001) show that infertility in India has increased among reproductive-age couples. It has risen from 13 per cent in 1981 to 16 per cent in 2001 among ever-married women [13]. It was observed that the infertility rate has declined between 1998–99 and 2005–06 [14]. Furthermore, another study from India found that about eight per cent of currently married women suffered from primary and secondary infertility, of which 5.8% per cent were secondary infertile [15]. This study also suggested that primary fertility decreases with age and was higher among younger women, while secondary infertility was higher among older women [15].

The causes of infertility issues are various, including social and biological factors. However, most of the studies agreed that around half of infertility among couples prevails due to anatomical, genetic, and immunological factors. Epidemiological studies identified the primary causes of infertility among women as menstrual disorders, diseases like obesity, thyroid diseases, diabetes, uterine factor, fallopian tubes, ovulation dysfunction, and cervical factor [16,17]. The concurrent review of literature on demographic and behavioural studies on infertility suggested that it is associated with the cohabitation age, couple’s lifestyle, mental stress, premarital sexual relationship, extramarital sex and obesity [18–21]. The research based on Indian women data found that infertility decreases with an increase in women’s age at marriage. Women who got married after 18 years of age were 81 per cent more likely to be infertile compared to those who married on or before the age of 18 years [21]. There are many preventable causes of infertility, such as sexually transmitted infections (STIs), health, lifestyle factors, and healthcare practices [22]. The infertility rate is higher in developing countries due to sexually transmitted infections and a lack of adequate and modern medical facilities [10,11]. Sexually transmitted diseases are a major cause of infertility. An increase in the number of sexual partners would lead to higher infertility [23]. Also, induced abortions may increase the risk of secondary infertility, particularly in women with subfertility, reflected in the occurrence of repeated miscarriages [24]. Many other preventable causes of infertility, such as environmental, socioeconomic and lifestyle-related factors increase infertility rate. These studies revealed that living environment of a couple such as frequent exposure to heat noise etc, have adverse effect on the couple’s reproductive life [25,26]. Further, pollution, water contamination, and chemical use in food and water also increase infertility.

Moreover, lifestyle factors such as obesity, diet and smoking have also an adverse effect on reproductive health [27]. People with high body mass index (BMI), that is, excess weight, have been shown to have a major impact on menstruation, infertility, miscarriage, pregnancy and labour [28,29]. Cigarette smoking, alcohol consumption, induced abortions, prior contraceptive use and higher body weight have been shown to have elevated the risk of suffering from infertility [30–33].

Worldwide, increasing maternal age can be seen as a common cause as women delay their marriage and childbearing age to get education and employment opportunities [14,21]. In the last few decades, the world has become more advance in many aspects, which has resulted in cultural, biological, social, and environmental change. These changes also contribute to a couple’s reproductive health, such as fertility outcomes. However, clinical studies suggested that over time, the prevalence of infertility has increased in developing countries [21,34]. It is interesting that the Infertility rate, as well as the fertility rate, is higher in developing countries than in developed countries. This may be happening because developing countries have not had adequate medical technology which can address infertility issues.

Infertility is generally defined as the inability of a couple to conceive despite one year of cohabitation without any contraceptive uses and exposure to pregnancy [6,9]. In clinical terms, infertility is a disease of the reproductive system due to failure to conceive even after being sexually active after one year or more of regular cohabitation without any contraceptive use [6,9]. Infertility may be classified into primary and secondary infertility, where primary infertility is the inability to bear any children, either due to the inability to conceive or the inability to carry a pregnancy to a live birth; and secondary infertility is the inability to bear a child after having an earlier birth. According to the World Health Organization (WHO), most infertile couples around the world suffer from primary infertility, which means that the woman has never conceived [6,9]. On the contrary, secondary infertility might occur at any time in a woman’s life after the first pregnancy, be it any outcome, a live birth, abortion or miscarriage. Some of the potential causes that are known, the most common factors among women are irregular ovulation leading to problems in the menstrual cycle, endometriosis and blockage of the fallopian tubes [9,21,35,36]. There is a dearth of literature, no literature to our best knowledge that provided recent estimates of infertility, including both primary and secondary infertility on Indian data in recent years is available. On one side fertility rate has been continuously declining in the country; on the other side, the use of contraception has not shown an encouraging trend [37]. During the same time, there has been increase in infertility-related reasons for not using contraceptives [38]. These patterns suggest a need to examine the trends of infertility in the recent past. Studies from India indicated that infertility levels were low and not likely to influence fertility levels at national or subnational [39]. However, infertility should be treated as a health issue, and therefore, the potential socio-demographic and lifestyle factors affecting both primary and secondary infertility need to be examined thoroughly. The socio-demographic covariates have been well established using large-scale survey data for India [15,21]. However, there is a dearth of literature that has attempted to unearth the role of lifestyle and sexual behavioural and non-communicable diseases such as thyroid, diabetes, cancer and hypertension using recent data.

## Materials and methods

The present study attempted to analyse the trends of primary and secondary infertility in India and states between 1992–93 to 2015–16. Further, we aimed to determine the socio-demographic and behavioural factors and diseases among women that may affect infertility. The study used a cross-sectional study design as its method of inquiry.

This study used secondary data from all four rounds of the National Family Health Survey (NFHS) data starting from NFHS-1 conducted in 1992–93, NFHS-2 (1998–99), NFHS-3 (2005–06) and the recent round NFHS-4 (2015–16) carried out in India. The four rounds were mainly used to obtain an overall trend of infertility over the years. The NFHS is a large-scale, multi-round survey conducted in a representative sample of households throughout India. The primary objective of the NFHS is to provide essential data on health and family welfare, as well as data on emerging issues in these areas. The clinical, anthropometric, and biochemical (CAB) component of NFHS-4 was designed to provide vital estimates of the prevalence of malnutrition, anaemia, hypertension, HIV, and high blood glucose levels through a series of biomarker tests and measurements. The present study includes a sample size of 76,648 in NFHS-1, 77,974 in NFHS-2, 124,385 in NFHS-3, and 482,763 in NFHS-4 currently married women aged 20–49 years.

### Ethical concerns

The analysis is based on secondary data available in the public domain for research; thus, no approval was required from any institutional review board (IRB). The survey agencies had conducted the fieldwork with prior consent from the respondents.

### Definition of outcome variables

The basic problem of infertility studies lies in the conceptualisation and definition due to the observed variations in the definitions adopted by different field researchers like social scientists, demographers, and clinical and medical scientists. The variations in the definitions occur mainly due to (1) the reference period used to establish infertility and (2) the categorisation of women who have experienced pregnancy and delivery but not a live birth. WHO defines infertility as the inability to conceive within two years of exposure to pregnancy. In addition, medical studies often use one year reference period of exposure to conceive. In demographic studies, it is common to use five years as an exposure time, and NFHS- survey uses a five-year reference period for infertility.

**Fig 1:**
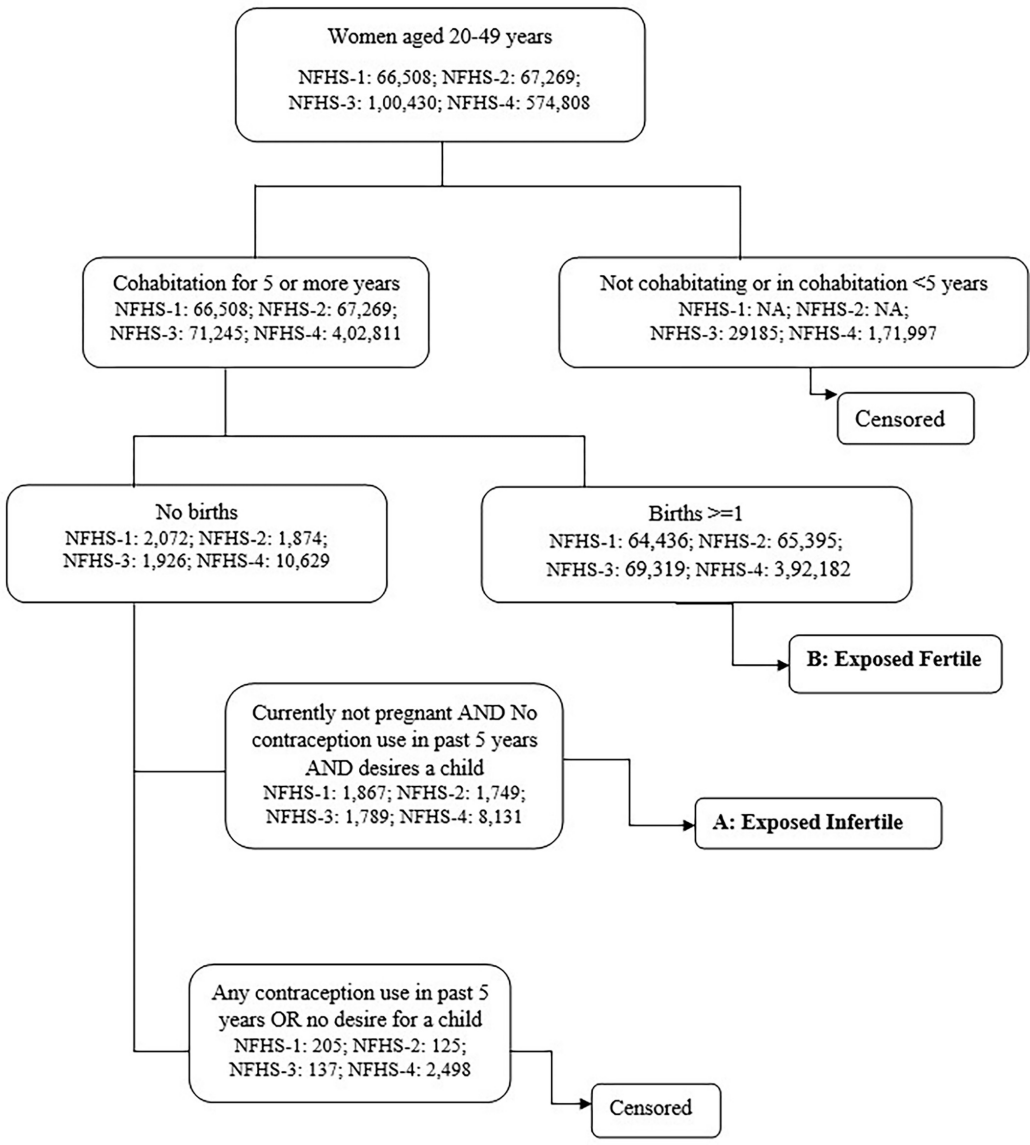
Measurement of primary infertility.

We adopted the standard definition proposed by Mascarenhas *et al*. (2012) [40], which defined **Primary infertility** as the absence of live birth for couples that have been in a union for at least five years, during which neither partner used contraception, and where the female partner expresses a desire for a child. The prevalence of primary infertility is calculated as the number of women in an infertile union divided by the combined number of women in fertile and infertile unions. Women in a fertile union have had at least one live birth and have been in a union for at least five years at the time of the survey (Fig 1).

**Fig 2:**
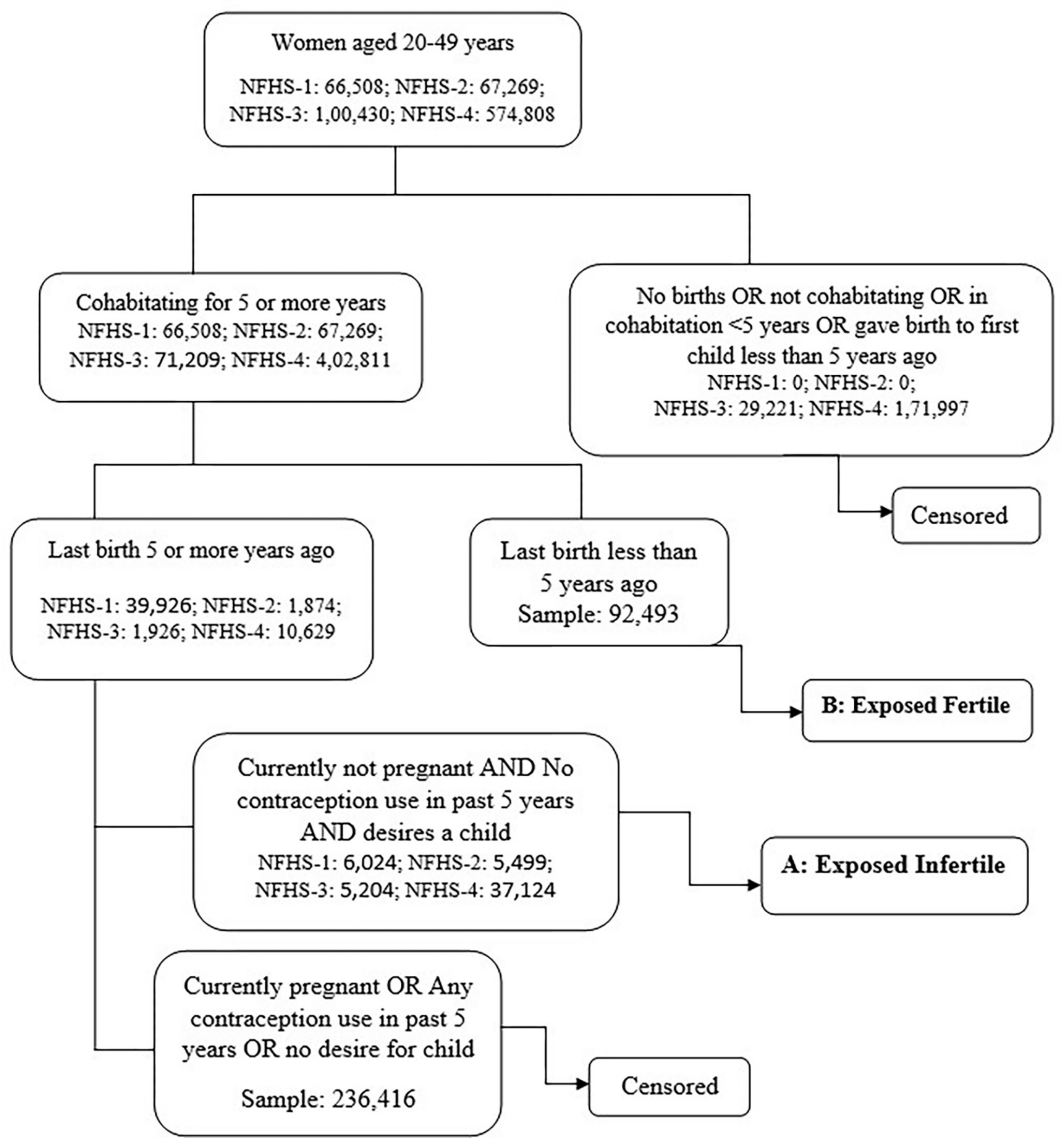
Measurement of secondary infertility.

**Secondary infertility** was defined as the absence of live birth for couples that have been in a union for at least five years since the female partner’s last live birth, during which neither partner used contraception, and where the female partner expresses a desire for a future child. The prevalence of secondary infertility is calculated as the number of women in an infertile union divided by the combined number of women in infertile and fertile unions. Women in a fertile union have had at least one live birth in the past five years and, at the time of the survey, have been in a union for at least five years following their first birth (Fig 2).

### Predictors from NFHS-4 database

**Fig 3:**
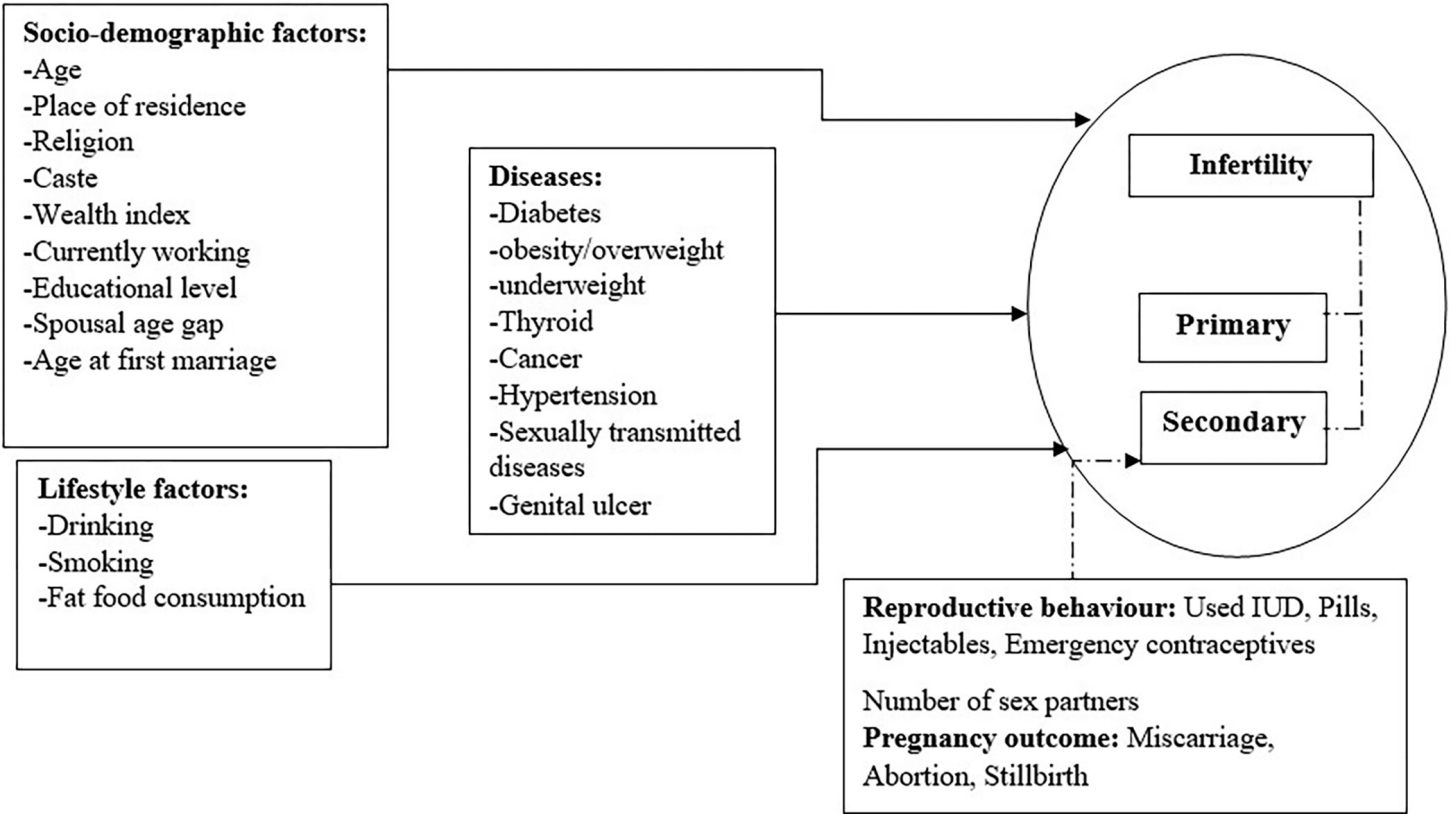
Conceptual framework for determinants of infertility.

This study used several potential socio-demographic, economic and health behaviour covariates to understand the determinants of infertility as portrayed in the conceptual framework (Fig 3). The classification is described in the following section-

The socioeconomic and demographic predictors include age group in years (20–24, 25–29, 30–34, 35–39, 40–44, 45–49), place of residence (urban, rural), religion (Hindu, Muslim, Christian, others), caste (SC, ST, OBC, Others), wealth index (poor, middle, rich), currently working (no, yes), educational level (no education, primary, secondary, higher), age at first marriage (below 18, 18–24, 25–29, 30–44, 45 and above), spousal age gap (wife is older than husband, 0–2, 2–5, 6 and above). The variable for spousal age gap is created by taking the difference between the variables age of husband and age of wife, and the was recoded as ‘Wife is older than husband” for all the negative values, ‘0–2’ for age gap of 0 to 2 years between husband and wife, ‘3–6’ for age gap of 3 to 6 years and ‘6+’ for age gap of more than six years between husband and wife. We have used the wealth index as a proxy of income which is a composite measure of a household’s cumulative living standard. NFHS survey does not collect direct information on the income and expenditure of households. The wealth index score of the household was constructed using principal component analysis (PCA). After that, we categorised it into five quantiles. In this study, the first two quantiles were categorised as poor, the third quantile was categorised as ‘middle’, and the fourth and five quantiles were categorised as ‘rich’.

Among the lifestyle predictors of infertility, we included drinking alcohol (no, yes), smoking (no, yes), body mass index (BMI) (below 18: underweight, 18 ≤ BMI <25: normal weight,25 ≤ BMI <30: overweight, BMI ≥ 30: obese), thyroid (no, yes), self-reported diabetes (no, yes), cancer (no, yes).

Further, the study included information on the sexual and reproductive behaviour of women/couples, such as number of sex partners (1, 2–5, 5–15, more than 15), have used IUD (no, yes), have used injectable (no, yes), have used pills (no, yes), have used emergency contraception (no, yes), had STI in last one year (no, yes), had a genital ulcer in last one year (no, yes).

### Analytical approach

We used a t-test to calculate the proportional change in infertility rates from 1992 to 2016, considering the p-value at a 5% level of significance. The bivariate analysis included cross-tabulation and chi-square test of association was adopted to understand the extent of infertility among Indian couples from different socioeconomic groups, different levels of lifestyle and reproductive behaviour in 2015–16. Finally, we applied a multivariate logistic regression model on primary and secondary infertility outcomes and presented adjusted odds ratios (AOR).

## Results

### Trends of primary and secondary infertility between 1992–93 and 2015–16

**Fig 4:**
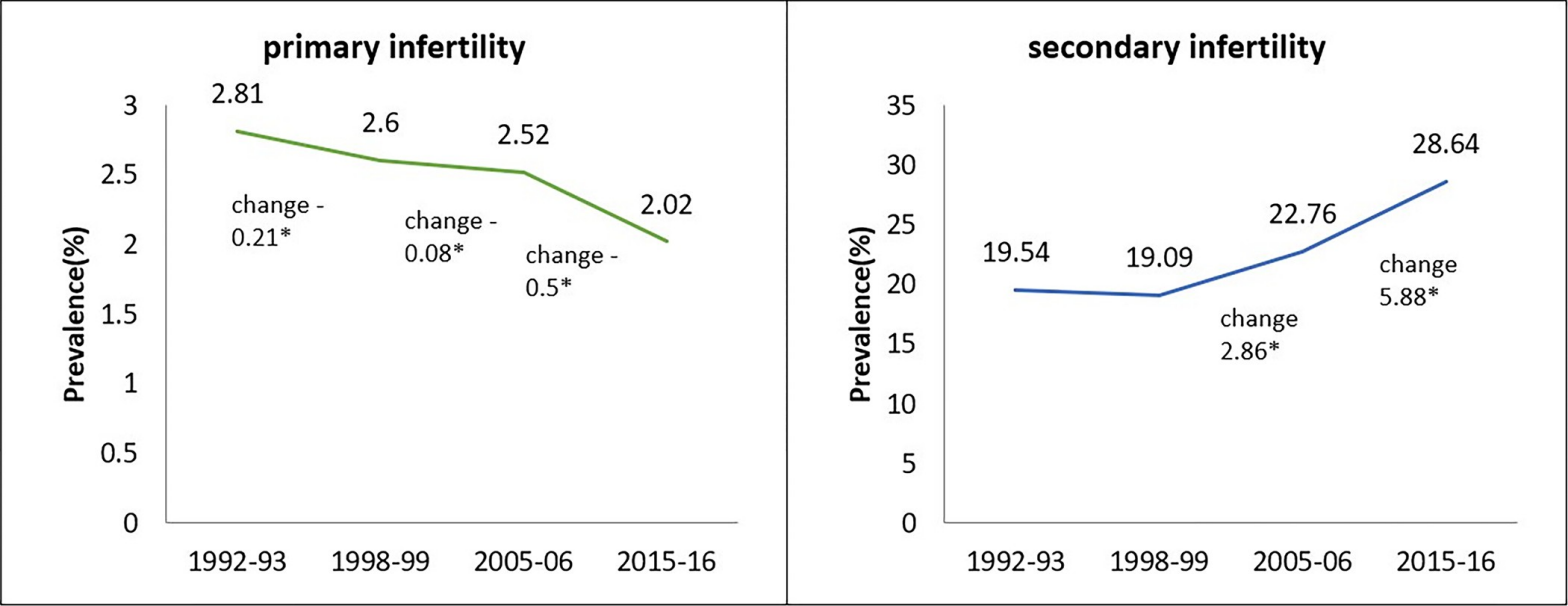
Trends of primary and secondary infertility in India, 1992–2015. **Note**- *: Proportion change 5% level of significance.

The trend of primary infertility depicts a gradual decrease over time, as depicted in Fig 4. It was around 2.8% in 1992–93 and significantly declined to 2.6% in 1998–99, remained at the almost same level of 2.5% in 2005–06 and then declined to 2% during 2015–16. On the other hand, the rate of secondary infertility has been significantly increasing from 1992–93 till 2015–16. It was around 19.5% in 1992–93, which remained almost the same in 1998–99, further increased by 2.9 percentage points in 2005–06. However, secondary infertility significantly increased by 5.9 percentage points in the recent decadal period of 2015–16 and reached 28.6% (Fig 4).

**Fig 5:**
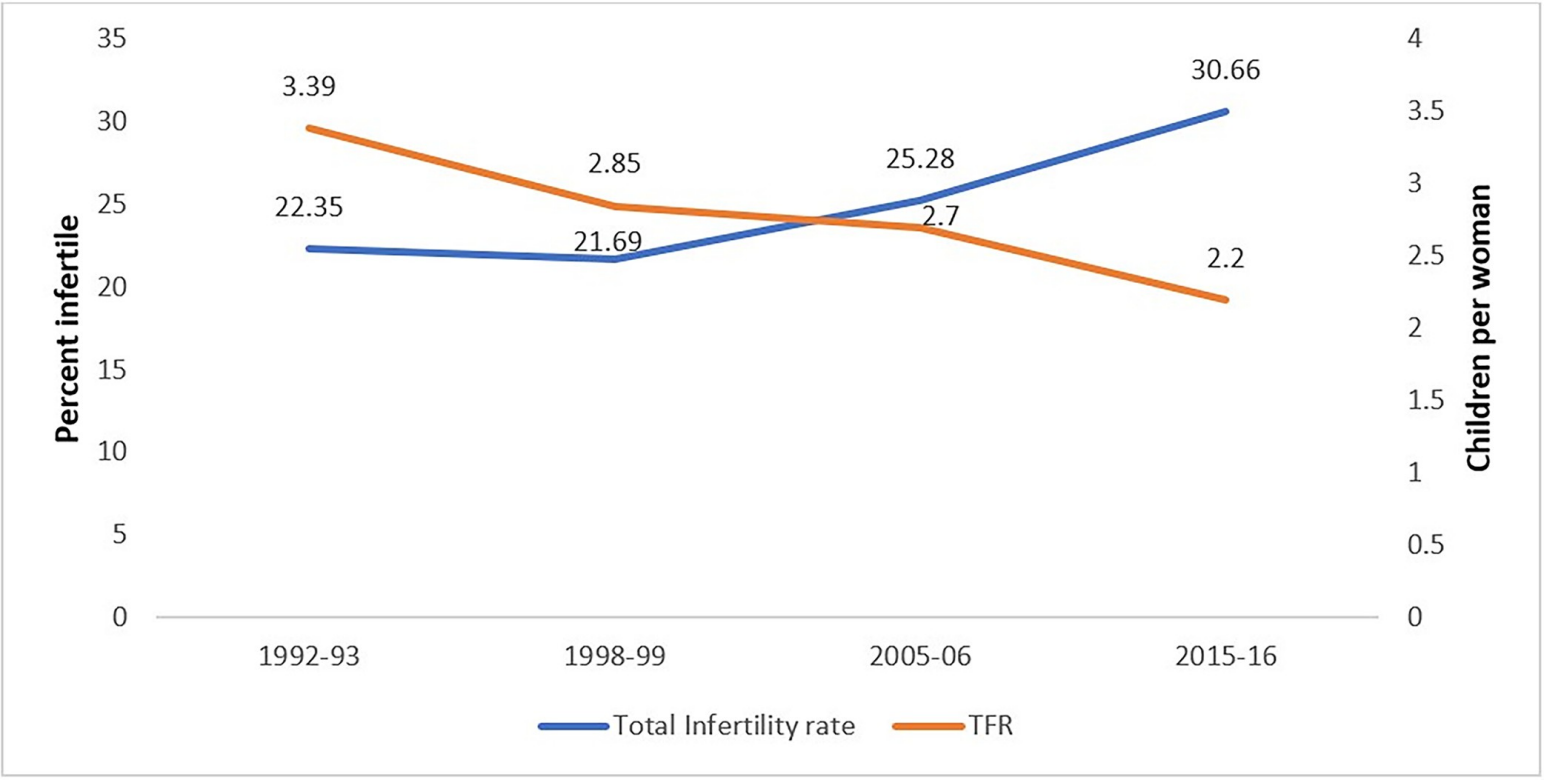
Trends of total fertility rate and infertility prevalence in India.

Overall infertility rate has increased from 22.4% in 1992–93 to 25.3% in 2005–06 and then increased to 30.7% in 2015–16 (Fig 5). This graph shows the infertility trend with total fertility rate (TFR), both declined considerably until 1998–99, but infertility increased rapidly coupled with a further decline in TFR between 2005–06 and 2015–16.

**Fig 6:**
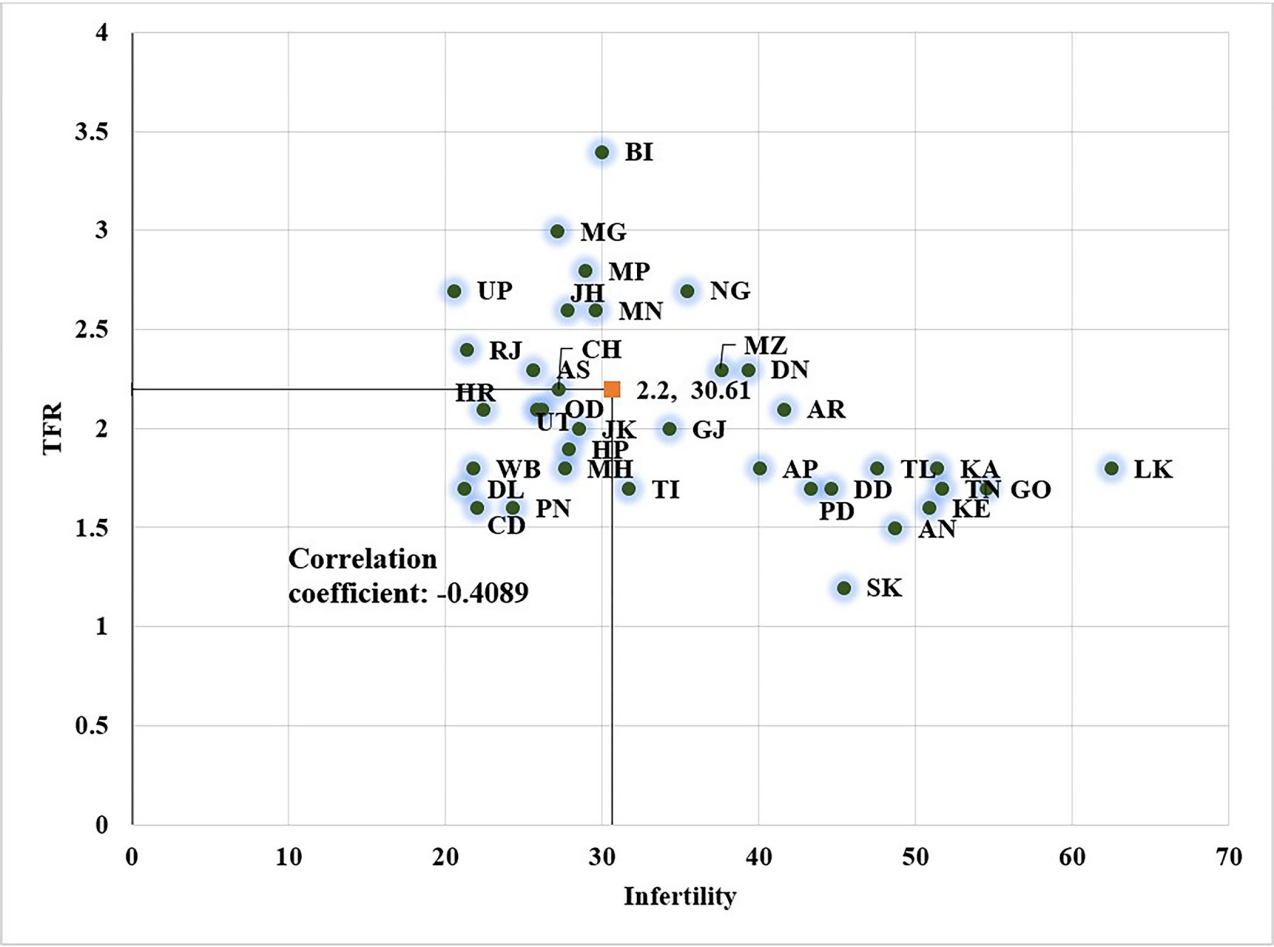
Association between total fertility rate and infertility in states of India, 2015–16.

This finding indicates that infertility can be a cause of TFR decline in certain regions. Therefore, we plotted state-level TFR and total infertility in the scatter plot (Fig 6). The states, namely- Goa, Kerala, Karnataka, Tamil Nadu, Telangana, Sikkim, Delhi, Himachal Pradesh and UTs- Lakshadweep, Daman and Diu, Andaman & Nicobar are having a high prevalence of infertility (above 20%) and low fertility rate (below 2). In contrast, states like Bihar, UP, Jharkhand, and the north-eastern states of Meghalaya, Nagaland and Manipur had both high infertility and fertility levels. Overall, the prevalence of primary infertility was higher (2%) in the states/UTs having TFR below replacement level, and secondary infertility was higher (28.6%) in the states/UTs having a medium level of infertility (Fig 6).

#### Effect of socioeconomic and demographic factors on primary and secondary infertility

Primary and secondary infertility were both associated with women’s age. The prevalence of primary infertility decreased with age, with the age group 20–24 having the highest prevalence (3.7%), whereas the prevalence of secondary infertility increased with age, with the age group 45–49 having the highest prevalence (89%). Primary infertility is estimated highly prevalent in urban areas than in rural areas (Urban: 2.13%; Rural: 1.89%). A similar pattern was observed for secondary infertility (Urban: 33.6; Rural: 26.5). Among the religious groups, Christians had the highest infertility level of both types, with 2.5% primary and 37.3% secondary. Furthermore, the prevalence of primary infertility was higher among STs (2.3%) and SCs (2%), while the prevalence of secondary infertility was higher among OBCs (30%). A higher prevalence (36.2%) of secondary infertility among persons from affluent households as compared to 22.6% among persons from poor wealth quantile families. Secondary infertility was observed to be significantly higher among working women (33%). Looking at the educational status of women, both primary and secondary infertility was the highest among women with high education (Primary: 2.5%; Secondary: 32%). Women who got married after the age of 30 years had a very high prevalence of both primary (8.3%) and secondary (around 53%) infertility. Secondary infertility was the highest among women who were six or more years older than their husbands, i.e. approximately 48% and primary infertility was more common among women who were 3 to 5 years older than their husbands, i.e. about 3.4% (**Table 1**).

**Table 1:** Prevalence for primary and secondary infertility according to socio-economic factors.

*Variables*		*Primary infertility (%)*	*Chi-square test (p-value)*	*Secondary infertility(%)*	*Chi-square test (p-value)*
**Current age**	**20–24**	3.67	<0.01	13.9	<0.01
** **	**25–29**	2.26		11.6	
** **	**30–34**	1.97		20.2	
** **	**35–39**	1.81		39.9	
** **	**40–44**	1.50		68.7	
** **	**45–49**	1.57		89.0	
**Place of residence**	**Urban**	2.13	0.08	33.7	<0.01
** **	**Rural**	1.89		26.6	
**Religion**	**Hindu**	1.99	<0.01	29.8	<0.01
** **	**Muslim**	1.90		22.9	
** **	**Christian**	2.47		37.3	
** **	**Others**	1.38		23.6	
**Caste**	**SC**	2.03	<0.01	25.7	<0.01
** **	**ST**	2.29		26.1	
** **	**OBC**	1.89		30.1	
** **	**Others**	1.94		29.7	
**Wealth index**	**Poor**	2.01	<0.01	22.6	<0.01
** **	**Middle**	1.98		30.5	
** **	**Rich**	1.93		36.2	
**Currently working**	**No**	2.00	0.72	26.9	<0.01
** **	**Yes**	1.86		33.1	
**Educational level**	**No education**	1.83	<0.01	30.0	<0.01
** **	**Primary**	1.79		26.2	
** **	**Secondary**	2.06		27.5	
** **	**Higher**	2.51		32.1	
**Spousal age gap**	**Wife older 6+**	1.65	<0.01	48.3	<0.01
** **	**Wife older 3–5**	3.42		35.1	
** **	**Almost same age 0–2 a**	2.02		23.8	
** **	**Husband older 3–5**	1.95		26.5	
	**husband older 6+**	1.97		29.0	
**Age at first marriage**	**below 18**	2.32	<0.01	53.2	<0.01
** **	**18–25**	3.37		56.5	
** **	**25–30**	9.44		66.1	
** **	**30–45**	16.16		97.7	
** **	**45+**	2.19		56.3	
**Sample**		**402526**	** **	**129618**	

The multivariate logistic regression model confirmed the impact of socioeconomic and demographic predictors on infertility. A woman’s age positively affects both types of infertility (adjusted odds ratio (AOR):1.41 for primary and 1.12 for secondary infertility). Age at marriage was the most significant factor affecting primary and secondary infertility. Women marrying between ages 30–45 years were 7.8 times and 4.6 times more likely to be primarily and secondarily infertile than women married before the age of 18. Women with higher levels of education were more likely than women with lower levels of education to experience primary infertility (AOR: 1.2), implying that the likelihood of being infertile rises as education levels rise. Primary infertility declines as household wealth increases. Additionally, working women had higher likelihood of secondary infertility than non-working women, making them the group with the highest risk. Primary (AOR: 1.163) and secondary infertility (AOR: 1.26) were more prevalent among Christian women than in other women. Women living in rural areas were 0.956 and 0.747 times less likely to be primarily and secondarily infertile, respectively (**Table 2**).

**Table 2:** Binary logistic multivariate regression model for primary and secondary infertility according to socio-economic factors.

		*Odds ratio (Confidence interval)*	
*Variables*		*Primary infertility*	*Secondary infertility*
**Current age**		1.405**(1.114,1.690)	1.201***(1.198,1.204)
**Place of residence**	**Urban ®**		
** **	**Rural**	0.956*(0.91,1.005)	0.747***(0.726,0.767)
**Religion**	**Hindu ®**		
** **	**Muslim**	0.967 (0.902,1.038)	0.749***(0.723,0.776)
** **	**Christian**	1.163***(1.067,1.268)	1.26***(1.211,1.312)
** **	**Others**	0.832***(0.741,0.934)	1.181***(1.113,1.253)
**Caste**	**SC ®**		
** **	**ST**	1.222***(1.137,1.313)	1.314***(1.263,1.367)
** **	**OBC**	0.947*(0.888,1.01)	1.238***(1.194,1.283)
** **	**Others**	0.874***(0.813,0.938)	1.269***(1.22,1.321)
**Wealth index**	**Poor ®**		
** **	**Middle**	0.946*(0.891,1.004)	1.474***(1.426,1.522)
** **	**Rich**	0.864***(0.821,0.909)	1.818***(1.768,1.869)
**Currently working**	**No ®**		
** **	**Yes**	0.978 (0.864,1.106)	1.399***(1.308,1.497)
**Educational level**	**No education ®**		
** **	**Primary**	0.92**(0.858,0.986)	0.851***(0.82,0.883)
** **	**Secondary**	1.019 (0.968,1.072)	0.859***(0.836,0.883)
** **	**Higher**	1.214***(1.113,1.324)	0.982 (0.933,1.033)
**Spousal age gap**	**Wife older 6+ ®**		
** **	**Wilfe older 3–5**	1.029 (0.422,2.512)	0.637**(0.407,0.997)
** **	**Almost same age 0–2 a**	0.775 (0.381,1.575)	0.443***(0.319,0.616)
** **	**Husband older 3–5**	0.706 (0.348,1.432)	0.509***(0.367,0.707)
	**husband older 6+**	0.758 (0.375,1.529)	0.59***(0.427,0.816)
**Age at first marriage**	**below 18 ®**		
** **	**18–25**	1.184 (0.875,1.604)	1.261**(1.055,1.506)
** **	**25–30**	3.856***(2.118,7.018)	1.824**(1.075,3.094)
** **	**30–45**	7.837***(3.748,16.387)	4.649***(1.574,13.736)
** **	**45+**	1.558**(1.096,2.216)	1.094 (0.899,1.331)
**Sample**		**402526**	**129618**

Note: ®: Reference category ***: 99% significance level **: 95% significance level.

### Effects of lifestyle factors, diseases and reproductive behaviour of married women on infertility

The effect of lifestyle factors, diseases, and reproductive behaviour was estimated after adjusting for socioeconomic and demographic factors (Table 3). We observed that primary and secondary infertility were more prevalent among women who drink alcohol, i.e. 2% and 37.4%, respectively. Secondary infertility was significantly higher (56.3%) among women who smoked. Infertility increases with the number of sex partners; primary and secondary infertility were more common among women who had more than one partner. Both primary and secondary infertility was the highest among obese women, i.e. 2.3% and 46%, respectively. Thyroid, diabetes and cancer were significantly associated with both secondary and primary infertility. As 3.2%, 2.7% and 5.7% of women were primarily infertile among those who had thyroid, diabetes and cancer, respectively. Further, 51.7%, 66.3%, 64.6% of women were secondarily infertile among those who had thyroid, diabetes, and cancer. Women, who had used intrauterine devices (14.8%), injectables (9.5%), oral pills (9.3%) and emergency contraceptives (7.9%) as family planning methods had significant associations with secondary infertility.

**Table 3:** Prevalence for primary and secondary infertility according to lifestyle factors, diseases and reproductive behavior.

*Variables*		*Primary infertility*	*Chi-square test (p-value)*	*Secondary infertility*	*Chi-square test (p-value)*
**Drinking alcohol**	**No**	1.97	0.09	28.5	<0.01
** **	**Yes**	2.00		37.4	
**Smoking**	**No**	1.97	0.59	28.6	<0.01
** **	**Yes**	1.11		56.3	
**Total number of sex partners**	**1**	1.83	<0.01	27.3	<0.01
** **	**More than 1**	4.89		35.3	
**BMI**	**Underweight**	1.80	0.12	20.3	<0.01
** **	**Normal weight**	1.94		26.7	
** **	**Overweight**	2.00		39.7	
** **	**Obese**	2.30		46.1	
**Thyroid**	**No**	1.93	<0.01	28.1	<0.01
** **	**Yes**	3.16		51.7	
**Diabetes**	**No**	1.95	<0.01	28.0	<0.01
** **	**Yes**	2.68		66.3	
**Cancer**	**No**	1.96	0.02	28.5	<0.01
** **	**Yes**	5.67		64.6	
**Had STI in last 1 year**	**No**	1.94	0.52	28.3	0.99
** **	**Yes**	2.71		30.8	
**Had genital ulcer in last 1 year**	**No**	1.93	0.21	28.1	0.93
** **	**Yes**	2.88		33.3	
**Have used IUD**	**No**	NA	NA	29.0	<0.01
** **	**Yes**	NA		14.8	
**Have used Injectables**	**No**	NA	NA	28.7	<0.01
** **	**Yes**	NA		9.5	
**Have used Pills**	**No**	NA	NA	30.8	<0.01
** **	**Yes**	NA		9.3	
**Have used Emergency contraception**	**No**	NA	NA	28.6	<0.01
** **	**Yes**	NA		7.9	
**Sample**		**402526**	** **	**129618**	** **

The adjusted multivariate logistic model, after controlling for the socioeconomic factors, shows that women who drink alcohol are more likely to be primarily (AOR: 1.12) and secondarily infertile (AOR: 1.52, than women who never drink. Women who smoke were 1.1 times more likely to develop primary infertility and 1.97 times more likely to develop secondary infertility than women who did not smoke. With the increase in the number of sex partners, the odds of developing primary infertility increased by 1.27 times and that of developing secondary infertility increased by 1.18 times. Obese women were around 1.11 and 3.16 times more likely to develop primary and secondary infertility, respectively than normal-weight women. For women with thyroid, the odds of developing primary and secondary infertility increased to 1.41 and 2.34 times, respectively. Women with diabetes had a higher risk of primary infertility (1.23 times) and secondary infertility (4.27 times). Women having cancer had the odds of developing primary infertility by 1.68 times and secondary infertility by 3.69 times. The use of intrauterine devices, injectables, oral pills and emergency contraception as contraceptive methods by women were 0.44, 0.22, 0.27 and 0.26 times less likely to be secondarily infertile than those who do not use (Table 4).

**Table 4:** Binary logistic multivariate regression model for primary and secondary infertility according to lifestyle factors, diseases and reproductive behavior.

		*Odds ratio (Confidence interval)*	
*Variables ©*		*Primary infertility*	*Secondary infertility*
**Drinking alcohol**	**No ®**		
** **	**Yes**	1.116*(0.982,1.268)	1.522***(1.425,1.625)
**Smoking**	**No ®**		
** **	**Yes**	1.104 (0.773,1.577)	1.968***(1.689,2.294)
**Total number of sex partners**		1.269***(1.145,1.406)	1.179***(1.108,1.256)
**BMI**	**Underweight ®**		
** **	**Normal weight**	1.003 (0.941,1.069)	1.41***(1.362,1.46)
** **	**Overweight**	1.038 (0.962,1.121)	2.429***(2.329,2.534)
** **	**Obese**	1.114**(1.003,1.238)	3.162***(2.978,3.357)
**Thyroid**	**No ®**		
** **	**Yes**	1.412***(1.246,1.601)	2.338***(2.162,2.53)
**Diabetes**	**No ®**		
** **	**Yes**	1.233***(1.062,1.432)	4.258***(3.867,4.689)
**Cancer**	**No ®**		
** **	**Yes**	1.678**(1.075,2.621)	3.687***(2.863,4.748)
**Had STI in last 1 year**	**No ®**		
** **	**Yes**	1.113 (0.803,1.542)	1.001 (0.83,1.209)
**Had genital ulcer in last 1 year**	**No ®**		
** **	**Yes**	1.193 (0.906,1.571)	0.993 (0.847,1.164)
**Have used IUD**	**No ®**		** **
** **	**Yes**		0.44***(0.407,0.476)
**Have used Injectables**	**No ®**		
** **	**Yes**		0.219***(0.171,0.282)
**Have used Pills**	**No ®**		
** **	**Yes**		0.268***(0.253,0.283)
**Have used Emergency contraception**	**No ®**		
** **	**Yes**		0.256***(0.169,0.39)
**Sample**		**402526**	**129618**

Note: ®: Reference category ***: 99% significance level **: 95% significance level.

©: Socio-economic variables were controlled (age, residence, religion, caste, wealth index, currently working, education, spousal age gap, age at first marriage).

## Discussion

The present study investigates the primary and secondary infertility considering the currently married women aged 20–49. The analysis did not include the 15–19 year group, ensuring that the phenomenon of adolescent sterility did not dilute the findings of the study. Our findings confirm that primary infertility has decreased; however, secondary infertility has increased, leading to an overall increase (10%) over time. It was previously shown that primary infertility dropped from 1998 to 2006 (21); however, no studies have investigated secondary fertility over time. Demographic health survey (DHS) comparative report (2004) had shown the estimate of secondary infertility in Southeast Asia as 23.5% [2]. Goa had the highest level of infertility in this study, followed by the southern Indian states, all of which experienced low fertility rates (TFR for Goa = 1.3 in 2019–20) [41]. The lifestyle risk factors, including smoking and drinking alcohol and the related disease, that is, diabetes, are also highly prevalent in Goa and some of the southern states which may explain the high levels of infertility in these states. Studies have shown that southern states have more early ages of female sterilisation and low fertility than northern states [42,43]. For instance, in a northern state like Bihar, female sterilisation prevalence is 34.8%, whereas, in the South, Kerala has 46.6% [41]. The total fertility rate is decreasing, and there is a perception that infertility is a cause of this declining infertility. When looked at the state level, the majority of the states with high infertility rates have fertility levels that are lower than or equal to the replacement level. It is interesting to note that the infertility rate among women over 30 at the time of their first marriage is higher than that of women under 30. Even from the literature [21], it emerged that age at marriage plays a significant role in causing infertility. Infertility is strongly and positively associated with age at first marriage. The findings additionally highlighted the importance of the spousal age gap and infertility. Previously, it was found that as the age gap between spouses increased, fertility rates declined [44]. Infertility is also prevalent among urban women, which might be due to a change in lifestyle or a later age at first marriage. Working women have a high rate of infertility, which must be primarily due to a stressful work environment, which has a significant impact on the menstrual cycle. Stressful life events like work stress and family pressure are associated with menstrual disorders [45], which lead to polycystic ovarian syndrome or disorder, and ultimately result in infertility [46,47].

The trend of smoking and drinking alcohol is more prevalent in urban areas, and this kind of lifestyle affects women in a way that later on affects their pregnancy, which has been previously stated in a study [30] conducted in the United States. Obesity is a vital factor affecting polycystic ovarian syndrome (PCOS), which is an important cause of infertility. Obese and overweight women tend to be more infertile as compared to normal or underweight women [27,29]. Diet also plays an important role nowadays as in today’s lifestyle women tend to consume more junk food which is not suitable for health and increases weight. This is confirmed by the finding that women who consume fatty foods like meat, eggs, and fried food tend to be more infertile than women who stay away from fatty food. Among the lifestyle factors, the number of sex partners as a factor is of relevance; that is, with an increasing number of sex partners, infertility increases. Thus lifestyle factors, including nutrition, weight, smoking, alcohol consumption etc., have an adverse impact on the reproductive health of women [48]. In order to delay pregnancies or even before marriage when women do not want to get pregnant, they tend to use oral contraceptive pills, emergency contraceptives or injectables. Now, these tend to affect the fertility of women, as also evident from the literature [27,29,30,48], and the findings also confirm so. Women who’ve had miscarriages and abortions tend to be more secondarily infertile. The thyroid has a significant association with female infertility, this can be related to the fact that obese women tend to be more infertile [27], and studies have shown that weight reduction has resulted in significant improvement in ovulation and pregnancy [49].

Age at marriage has risen, leading to family planning at a later stage. Lifestyle changes in recent times are becoming worse and unhealthy, including increasing age at marriage, an increasing number of working women who delay pregnancy, rising alcohol and tobacco consumption, a sedentary lifestyle together with fast food consumption, and disturbing levels of obesity [29,50]. More educated women are more likely to postpone marriages and childbirth. They are also expected to opt for smaller family sizes as they are busy with their careers and work commitments.

The present study has several strengths which include, the estimation of infertility utilizing large-scale population-based survey data. This gives an overview of the national scenario of the prevalence of infertility which is otherwise not available. Infertility, both primary and secondary, has been defined as robustly as possible. Since not much literature is available on infertility in the Indian context, this study is a major contribution to literature, by increasing the reliability and validity of the prevalence. However, there are certain limitations which can be taken note of and work in future research. The biggest limitation of the study is that here infertility is demographically defined, which is based on assumption and is not backed by medical tests and evidence. Continuous exposure to the risk of pregnancy which includes data on marital status, abstinence, coital frequency and timing, contraceptive use, termination of pregnancy and the partner’s presence or absence for all women for the entire period under consideration, is difficult to measure based on whichever definition is used. It was also not possible to consider factors like physical activity, mental health and sleep patterns since the survey does not collect information on these.

Infertility is underestimated as it is based only on currently married women; there might be couples who are unable to have children who may break their marriages or union. Other biases arise because women who gave birth to a child during the period of measurement may subsequently have become infecund [2] or because women who have not born a child during that period may have had an unreported miscarriage or an induced abortion, been temporarily separated from their partner, been ill, failed to report contraceptive use, or stopped having intercourse. Infertility measures are based on the fertility of couples at the end of the childbearing period, which does not reflect recent trends in infertility. The measurement of primary infertility is complicated, as there might be some women who do not desire to have any children and use contraception to achieve this desire, while other women want children but use contraception to postpone their first birth. Some of these women, when they are ready to have children, tend to find that they cannot be pregnant and become involuntarily infertile. The study does not take into account the desire for a child, and this might underestimate the level of involuntary infertility. Further studies can be carried out based on couple infertility, and the contribution of both males and females can be checked equally. A more concrete definition could be formulated based on continuous exposure to the risk of pregnancy.

## Conclusion

Although primary infertility has decreased over time, from the present study, it is evident that a sharp increase has occurred in secondary infertility among Indian couples, particularly between 2005–06 and 2015–16. This increasing trend raises concerns among health providers and researchers. The state of Goa, southern states and union territories, including Lakshadweep, Daman & Diu, showed a higher infertility rate in 2015–16. Overall, women from states with lower TFR levels reported higher infertility as compared to women from states with a high level of TFR. This further indicates that increasing infertility may be contributing to the declining fertility rates in India. Lifestyle factors affect both primary and secondary infertility in India. Eating healthy food and avoiding smoking and drinking alcohol are highly recommended as BMI and non-communicable diseases are important lifestyle factors for infertility. The problem of infertility is more common among women from underprivileged socioeconomic backgrounds.

### Policy implications

Our findings have important policy implications and can help in the identification and intervention of rising infertility. In India, the infertility issue is serious yet neglected, and it is frequently overlooked in public health discussions. The alarming trend of infertility necessitates the establishment of an infertility management chain comprised of trained doctors, counsellors, and health professionals who can provide information on cause and treatment at a reasonable cost. NGOs, self-help organisations, women’s organisations, ASHA, and the media can offer infertility-related awareness programmes at the household level.

Offering incentives and subsidised treatment may be beneficial in reaching out to the marginalised sections who cannot afford expensive and long-term treatment. Infertility clinics can be opened in high-risk areas to identify the vulnerable population. Young males can be a susceptible group for infertility due to drastic changes in lifestyle behaviour; therefore, there is a need to educate men about infertility causes and treatment. The government can establish a public provider to manage and treat infertility, as well as a testing centre and treatment options for sailors. Awareness campaigns and low-cost reproductive health services should be made available to people from all socioeconomic backgrounds. Therefore, we urge that infertility should be considered as an essential component of reproductive health policies and programmes.

## Abbreviations

WHO: World Health Organization
NHFS: National Family Health Survey
ICPD: International Conference on Population and Development
BMI: Body Mass Index
SC: Scheduled Caste
ST: Scheduled Tribe
OBC: Other Backward Classes
AOR: Adjusted Odds Ratio
TFR: Total Fertility Rate
PCOS: Polycystic Ovarian Syndrome
